# Syndemic geographic patterns of integrated diseases during the Libyan armed conflict; a new aspect for public health care intervention?

**DOI:** 10.3389/fepid.2025.1580437

**Published:** 2025-11-07

**Authors:** Mohamed Ali Daw, Abdallah H. El-Bouzedi, Saleh Ali Abumahara, Abdurrahman Khalifa Najjar, Nouri R. Ben Ashur, Alaa Grebi, Amnnh Mohammed Dhu, Emad Elsgair Alzahra, Ali Fathi Alkarghali, Shahid Husayn Mohammed, Raja Khalid Miftah, Najmuldin Abdulbasit Abdulsamad, Mohammed Saad Elbasha, Asawer Seifennaser Doukali, Nosieba Taher Elmhidwi, Esra Othman Albouzaidi, Said Emhamed Wareg, Mohamed Omar Ahmed

**Affiliations:** 1 Department of Medical Microbiology & Immunology, Faculty of Medicine, University of Tripoli, Tripoli, Libya; 2 Department of Statistics, Faculty of Science, Tripoli University, Tripoli, Libya; 3 Department of Surgery, Faculty of Medicine, Tripoli, Libya; 4 Department of Gynecology, Tripoli Medical Centre, Faculty of Medicine, Tripoli, Libya; 5 Faculty of Medicine, University of Tripoli, Tripoli, Libya; 6 Department of Biology, University of Nalout, Nalout, Libya; 7 Department of Microbiology & Parasitology, Faculty of Veterinary Medicine, University of Tripoli, Libya

**Keywords:** syndemic profiling, integrated diseases, armed conflict, Libya, TB/COVID-19, HIV/HCV, mortality/morbidity

## Abstract

**Introduction:**

Synedmic geographic analysis is new epidemiological tool used to implement crucial effective intervention to combat integrated diseases. This study aims to determine spatial patterns and geographic profiling of three concurrent diseases including TB/CPVID-19, HIV/HCV, and Mortality/Morbidity in Libya during the Libyan armed conflict.

**Methods:**

Geographic thematic mapping and spatiotemporal analysis were used to examine the syndemic geographic profiling of three integrated diseases including COVID-19 and TB, HCV/HIV, and Mortality and Morbidity during the Libyan armed conflict. The total number of notified TB and the cumulative number of COVID-19 cases, HIV and HCV cases, and Mortality and morbidity cases during the conflict period were reported. Such data were obtained at individual and geographic levels from each district involved in the armed conflict then analyzed and classified according to location, timing, and intensity of the Libyan armed conflict.

**Results:**

High co-occurrence of TB and COVID-19 was evident. The southern region (i.e., Sebha), Tripoli, and Benghazi consistently portrayed higher incorporation patterns of the two intertwined infections. Conversely, the western mountain region and the Southeast region exhibited a lower concordance during the pandemic period. The co-occurrence of HIV and HCV infections was clear all over the country. The highest condensation of the concomitant is in the Western region, particularly the western mountains, Zawia followed by Jufra and Ghat. Followed by the Eastern region, particularly Deana and Benghazi. This was less tense in the Southern and Med region municipalities. Mortality and morbidity show a visible syndemic geographic pattern. The highest density of these two concomitant patterns was Benghazi, Derna and, Ajdabia in the Eastern region and Sirt, Musrta, Baniwaled in the Western region and to a lesser extent in Zawia and Shati.This study highlights the need syndemic geographic patterns of integrated diseases to focus on wellbeing beyond standard health parameters. Clear decisions about prioritisation of health care to be provided based the geographic region in need.

## 1 Introduction

Geographic mapping is considered one of the most important public health tools in identifying spatial patterns of diseases across one or more geographic regions (1, 2). The Syndemics framework describes two or more co-occurring epidemics that synergistically interact with each other. Syndemic Such approaches have shown a measurable effect on health care and quality of life when applied to public health and clinical medicine. Syndemics render prevention and intervention programs more successful when addressing the multiple disorders and specific contextual vulnerabilities holistically, rather than viewing the disorders individually (3–5). A geographic syndemic framework is composed of two or more geographically co-occurring diseases that interact with each other. Such approaches garnered significant attention, and the focus has now shifted to the development of robust mixed methods to evaluate syndemics (6, 7). This resulted in great improvements in health outcomes and management of patients as those with diabetes and co-occurring depression and HIV and Tuberculosis. Such, successful approaches are rarely applied to diseased populations during armed conflicts (8, 9). They destroy health care systems and social infrastructures, create refugee populations, and spread infectious diseases.

These conflicts are known to be a disruptive process that sets in motion interactions between diseases and other conditions that increase war-related morbidity and mortality (10, 11). However, there remain enormous gaps in our knowledge about the relationship between armed conflict and health.” Hence then, studies are needed to highlight such an important concept.

Libya is one of the largest and richest countries in North Africa with the longest coast in the Mediterranean basin facing Europe. The country has been engulfed in internal conflict since 2011. This resulted in a high rate of injury, Mortality, and population displacement. The trends of injury, mortality, and the emergence of infectious diseases and their impact on the Libyan healthcare system have been well studied by Daw and his collaborators (12–15). The experience in Libya offers a great opportunity to study the interrelationships between diseases and armed conflict. Emphases should be directed towards observational study designs that go beyond statistical parameters to test causal inference.

In response to the complex challenges of delineating contingent causalities resulting from the armed conflict. Emphases should be directed toward observational study designs that go beyond statistical parameters to test causal inference (16, 17). Therefore, by examining war-related syndemic geographic patterns in various settings and periods, we can better understand the implications of such an approach for producing unmet public health needs. The scope of this study is aimed to focus on war-related geographic syndemic patterns in three various settings during the Libyan armed conflict. These include the interrelationship between; Tuberculosis and COVID-19, HCV and HIV infection, and mortality and morbidity.

## 2 Methods

### 2.1 Data source

This comprehensive national data collection was guided by our previously published studies and guidelines (18, 19). Libya is divided into three regions with twenty-two municipalities; East region (7 municipalities), West region (9 municipalities), and South region (6 municipalities) as previously published (12, 13) Data were collected from all the provinces in the three official regions of Libya during the conflict period.

#### 2.1.1 TB and COVID

To investigate the epidemiological association between TB and COVID-19 infections, we traced all the notified TB cases and the cumulative number of COVID-19 cases during the emergence of the COVID-19 pandemic in Libya. The time and spatial distribution of COVID-19 and TB cases were compared, and the correlation between both of them was determined. The spatial autocorrelation analysis was conducted by using open GeoDa software v1.2.0 (GeoDa Center for Geospatial Analysis and Computation, Arizona State University, Tempe, AZ) and creating thematic mapping, as previously described (20).

#### 2.1.2 HCV/HIV

The data collected consists of all the HCV and HIV infections newly reported throughout the country since January 2011. The data covered all the Libyan regions and the residence of each case was localized at the city and district levels. Laboratory diagnosis was carried out according to the procedures of the Libyan Central Laboratory. HCV testing was carried out by detecting HCV and HIV antibodies using an enzyme-linked immunosorbent assay (ELISA) and confirmed by immunoblot and/or polymerase chain reaction. Any person found to be positive and confirmed by these diagnostic tests is considered to be a case of infection (18, 21).

#### 2.1.3 Mortality and morbidity

Information was obtained from the National Death Registry offices in the three Libyan regions, and the Ministry of State for Families of Martyrs, Injuries, and Missing Persons (12.13]. Other sources of information were the local authorities in each region. Official government reports and reports of the Libyan Red Crescent were consulted, and accounts were obtained from eyewitnesses and combatants. The information included locations, event types, groups involved, fatalities, injuries, disabilities, demographics, and cause of death or injury.

The study covered individuals aged 15 years or more who have been confirmed as killed or injured as a direct result of the conflict from January 1, 2011. Civilian casualties were excluded. Inclusion criteria were death, injury, or disability as a direct effect of the armed conflict, and availability of information on the date, district, cause of death, and demographic characteristics (sex, age, and educational level) (11, 12).

#### 2.1.4 Geographic and syndemic analysis

Geo-data (Libya's map and the administrative municipalities' geographical borders) and patient data were used for geospatial analysis on grid maps. Geospatial syndemic analysis was carried out by applying the data of the individuals to the most recently updated electronic map of Libya and its districts showing the municipalities' borders, based on which distribution maps were constructed. The main spatial descriptive statistics (spatial mean center and spatial standard distance ± 2 standard deviations) were illustrated on these maps. The geographic data from each district involved in the armed conflict was classified according to the location, timing, and intensity of the Libyan armed conflict since 2011. Geospatial analysis of mortality and injury was analyzed at the district level as previously described (13, 14). ArcGIS 10.1 software, Environmental Systems Research Institute Inc., 1999 (ESRI Inc., Redlands, CA, USA) was used to create electronic maps. SPSS18.0 software (IBM Inc., Armonk, NY, USA) was used to analyze the data (22).

## 3 Results and data interpretation

### 3.1 Syndemic geographic patterns of tuberculosis and COVID-19

The SARS-CoV-2 epidemics overlap with endemic and seasonal diseases such as tuberculosis and other respiratory diseases and there is a significant concern that the emergence of COVID-19 is currently overlapping with these circulating pathogens (18, 19). This, however, is more complicated during the armed conflict. Thus the situation needs specific observations and high monitoring as both conditions could potentially lead to serious outcomes (20, 21). In this case, we aimed to assess the geographic syndemic framing of COVID-19 and tuberculosis in Libya.

**Figure 1:**
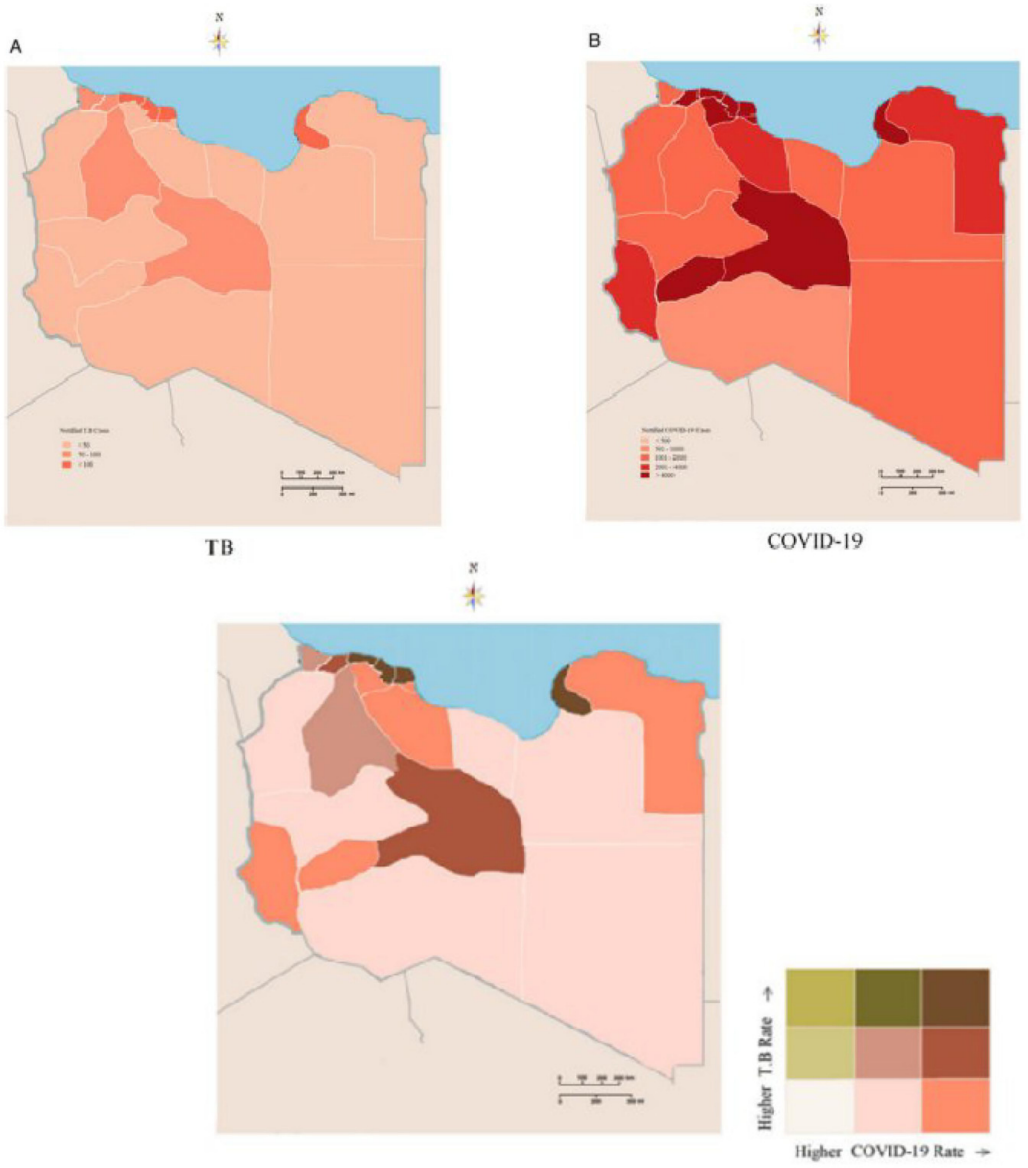
Syndemic geographic patterns of Tuberculosis and COVID-19 **(A)** Tuberculosis monoinfection **(B)** COVID-19 monoinfection **(C)** synedemic geospatial density of Tuberculosis and COVID-19.

Using thematic mapping as previously described (12, 13, 23), the number of notified TB and the cumulative number of COVID-19 cases were reported during the first twenty-eight epi-weeks of the pandemic spread as illustrated in Figures 1A,B. A total of 41,686 COVID-19 cases were documented, during the first twenty-eight epi-weeks. Only 75 cases were reported in the first eight weeks, followed by 466 and 1247 cases during the twelfth and sixteenth Epi-weeks respectively. On the twentieth epi-week (August) a sudden increase was noticed to reach the highest in September and October. During the same period, an average number of 177 TB cases were notified weekly during epi-weeks 1–8 and a mean number of 151 TB cases were notified weekly during the 28th epi-week.

The geo-epidemiological analysis of the two epidemics indicated a geographic integration between both of them as illustrated in Figure 1C. The map portrays what appears to be high co-occurrence in certain locations of the study area. The southern region (i.e., Sebha), Tripoli, and Benghazi consistently portrayed higher incorporation patterns of the two intertwined infections during the pandemic period. Conversely, the western mountain region and the Southeast region of the study area exhibited a lower concordance. This indicated the integration of COVID-19 and Tuberculosis both at concentration and interaction levels during the pandemic period. Therefore, in regions with high burdens of tuberculosis, maintaining a continuity of services and recovery programs should be a high priority to reduce the broader health impact of the pandemics (24, 25).

### 3.2 Syndemic geographic patterns of HIV and HCV

Hepatitis C virus (HCV) and human immunodeficiency virus (HIV), represent two highly prevalent chronic viral infections worldwide. In combination, these infections (HIV, HCV) increase the risk of morbidity and mortality when compared to mono-infection. Internationally, HIV/HCV coinfection is associated with a 6.7-fold increased morbidity and mortality. Despite advances in the treatment and prevention of HCV and HIV continue to constitute a considerable burden on health particularly in African and Middle Eastern countries (26, 27). These two viruses share the same transmission routes, and their co-occurrence depends on the presence of shared risk factors and community prevalence rates (28, 29). The presence of these multiple risk factors can interact with each other to increase the risk of infection transmission.

In Canada, a Cohort Study on the Characterization of HCV, and HIV Co-infections in a large population study concluded that the co-occurrence of infections and risk factors described suggests the presence of unique syndemics among individuals with HCV and HIV in British Columbia (28). In South Africa, geographic analysis has been used to analyze the consumption of alcohol, and intimate partner violence, among HIV infected women, indicating the need for geographically-tailored interventions (30). However, there is a notable gap in research on the geographic syndemic that examines these epidemics through a syndemics lens and analytic approach in low- and middle-income countries.

**Figure 2:**
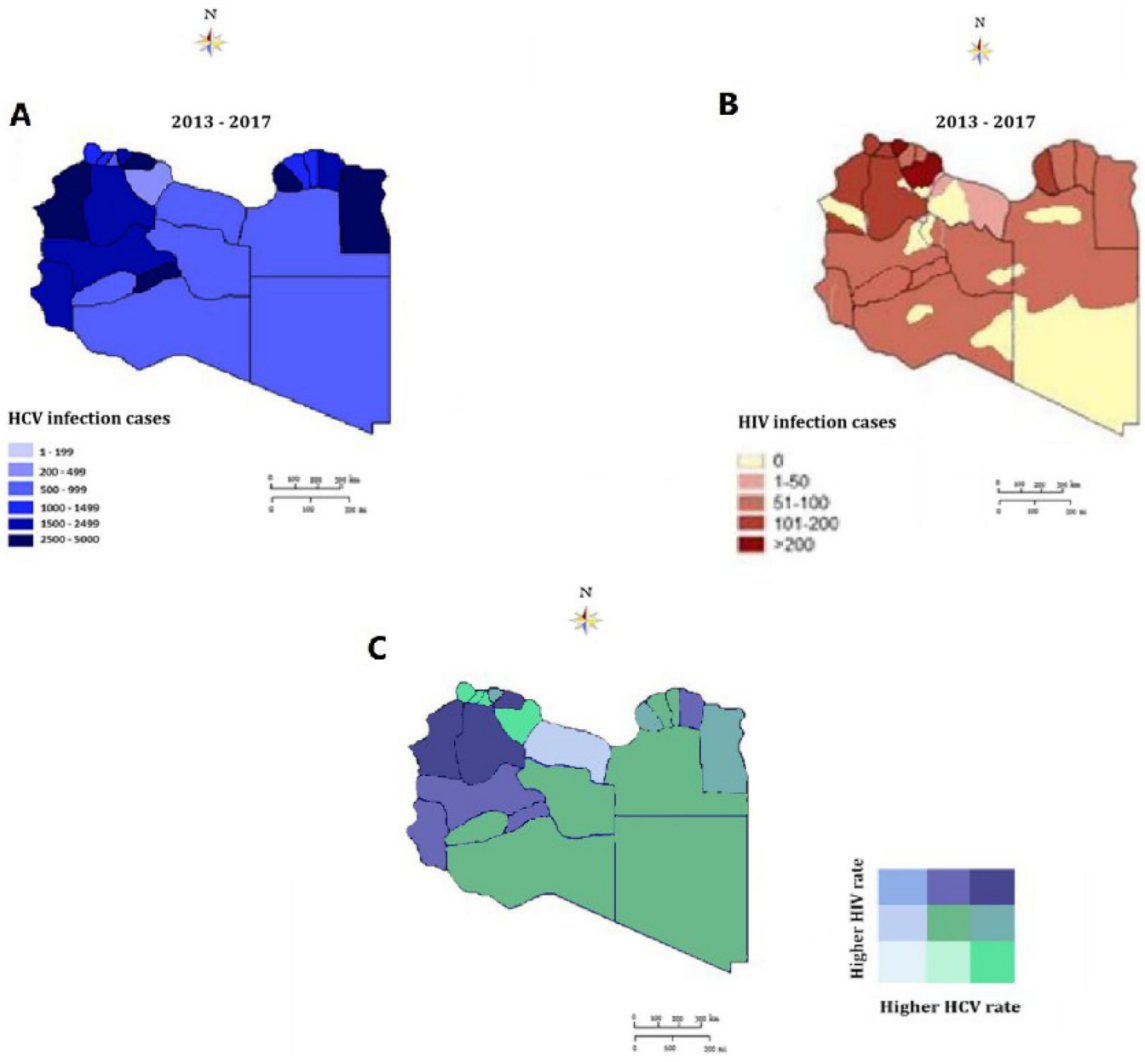
Syndemic geographic patterns of HIV and HCV; **(A)**, HCV mono-infection **(B)** HIV mono-infection, **(C)**–synedemic geospatial density of HIV/HCV coinfection.

The prevalence of HCV and HIV is more complicated in war-affected areas and data on the frequency and impact of concurrent infections on mortality risk are limited. This study was undertaken to analyze the syndemic geographic patterns of HIV/HCV coinfection during the Libyan armed conflict. During the Libyan armed conflict period the patients infected by HCV and or HIV were displayed and spotted on the Libyan map as illustrated in Figure 2. A total of 2,751 cases of Hepatitis C-infected individuals were reported. The geographic distribution was variable with the regions and within the municipalities of each region as shown in Figure 2A. The highest rate was reported in the Eastern region particularly Benghazi followed by Elbtanain, Aljabe Alagder, and Derna. Followed by the Western region bordering Tunisia and Algeria. The density is observed in the Western mountains, Zawara, and less extent in Aljfra, Wadi Shati, and Ghat. However, the spatial density of HCV was less in the Southern region and Meddle in the Western region (Figure 1A). The Geospatial distribution of HIV-infected cases during the same period is shown in Figure 2B. The highest rate of HIV was reported in Benghazi in the Eastern region, followed by Derna, and Albtnan. Musrata, Tripoli, and Zawara showed the highest density in the Western region followed by Aljabel Algarbi and Jufra, and to a lesser extent in the Southern region municipalities. The geographic integration of the two infections was analyzed throughout the study period. A clear syndemic geographic pattern of the Integrated Diseases was evident as shown in Figure 2C. The co-occurrence of both diseases was clear all over the country. The highest condensation of the concomitant was in the clear in the Western region, particularly the western mountains, Zawia followed by Jufra and Ghat. Followed by the Eastern region, particularly Derna and Benghazi. However, this syndemic geography was less tense in the Southern and Med region municipalities.

### 3.3 Syndemic geographic patterns of mortality and morbidity

Armed conflicts are a major cause of death all over the world. It is estimated that in the 20th century alone, sixty-two million civilians suffered war-related deaths, in addition to forty-five million combatant deaths (31). The consequences of such conflicts could last for a longer period, particularly within the Arab region which is more procumbent to these conflicts. The Interactions of morbidity and mortality during and after armed conflicts should have multiple and profound expressions that are rarely explored by researchers (32, 33). Libya has been provoked by armed conflict since 2011 which resulted in over 60 000 deaths and more than 135,000 injured. In this study, we determined the syndemics geographic pattern of morbidity and consequent morbidity during the Libyan armed conflict from 2011 to 2021. A total of 16,126 deaths and 42,633 injuries were recorded with complete information during the Libyan conflict from 2012 to 2017. Geospatial analysis was carried out by applying the data of the individuals to the most recently updated electronic map of Libya and its districts showing the municipalities' borders, based on which distribution maps were constructed.

**Figure 3:**
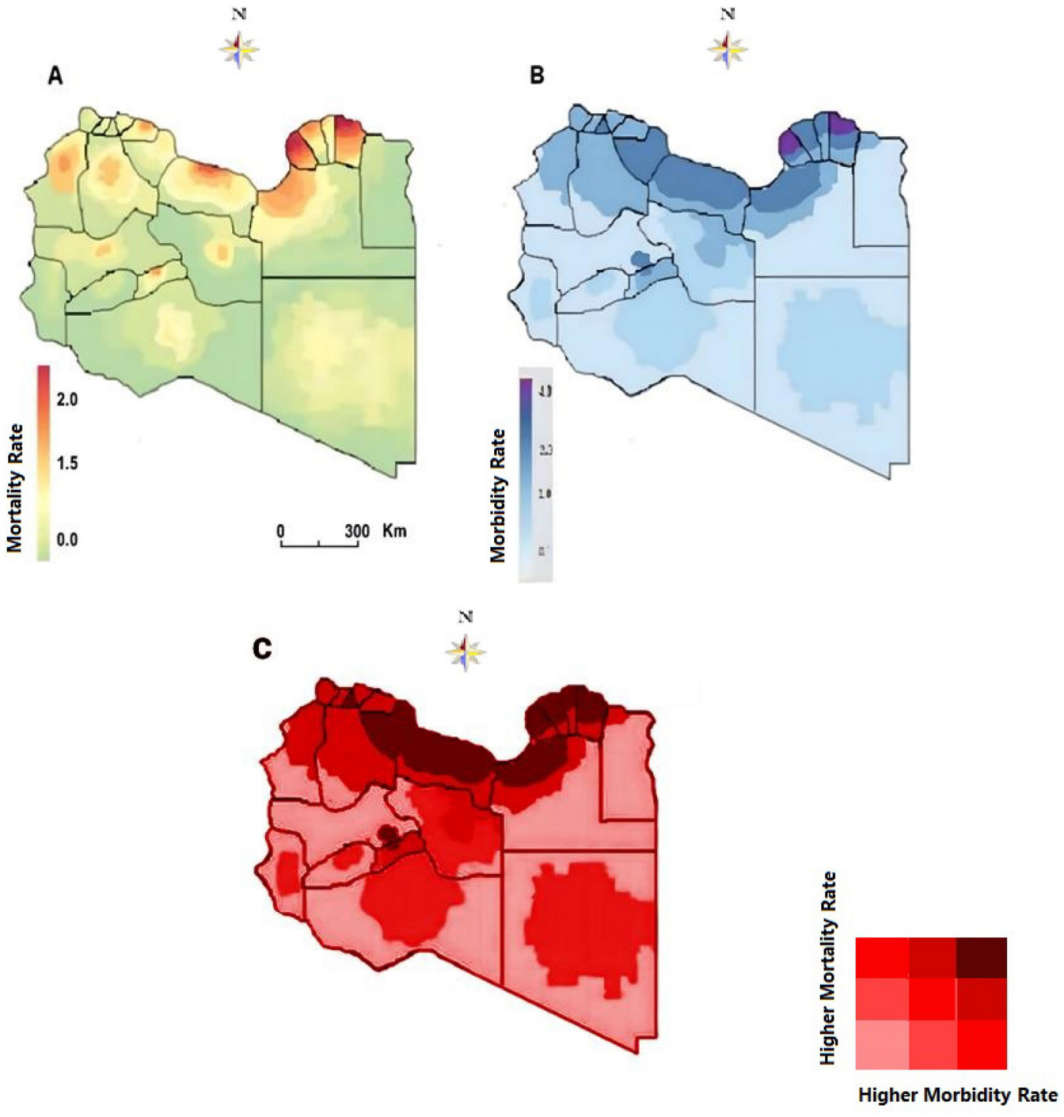
Syndemic geographic patterns of morbidity and mortality **(A)** mortality patterns **(B)** morbidity pattern **(C)** synedemic geospatial density of morbidity and morbidity.

Geographic patterns of mortality and mortality rates (per 1,000 population) varied substantially between regions and between parts of each region as illustrated in Figure 1. The morbidity varied greatly from one region to another and even among municipalities as shown in Figure 1A. The highest was reported in the Eastern region, particularly Benghazi, Derna, Aljabel Alagder, and Ajdabia followed by middle region municipalities including Musrta, Sirt Baniwaled, and Tarhona. Though it was less dense in the Western and Southern municipalities. The mortality patterns were geographically variable from one region to another it was less dense in the Southern region though it was dense in the Eastern region and to a lesser extent in the Western region as shown in Figure 1B. The highest rate of mortality was in Benghazi, Derna, sert, Baniwaled and Musrta. When the two parameters are geographically assigned together, they show a visible syndemic geographic pattern of mortality and morbidity as shown in Figure 3C. The highest density of these two concomitant patterns was Benghazi, Derna and, Ajdabia in the Eastern region and Sirt, Musrta, Baniwaled in the Western region and to a lesser extent in Zawia and Shati.

## 4 Discussion

As armed conflicts still going on and no signs that they will end, hence then expanding our understanding of syndemic geographic profiling of integrated diseases during conflicts should be a priority to improve public health efforts for war-affected populations (34, 35). In this study, we have examined three studies on TB/COVID-19, HIV/HCV, and mortality/morbidity during armed conflict to propose a syndemic geographic profiling that highlights the impact of wartime conditions on three integrated diseases. However, despite that the specific pathways of interaction and co-occurrence in each study discussed above occurred differently, the unifying thread between them is that the individuals studied are in armed conflict zone.

The synergistic interaction between integrated diseases can further aggregate the burden of the disease and subsequently impact the health quality within a population, particularly during the conflict timing (36). TB and COVID-19 have similar clinical symptoms and underdiagnoses and under-notification could be expected. This could lead to delays in treatment and diagnosis particularly for TB and thus an increase in mortality which may have an enormous impact on the number of people seeking and receiving healthcare for TB and COVID-19 (37, 38). Hence then a large-scale syndemic geographic profiling of TB and COVID-19 becomes a priority to intervene and implement the needed proper healthcare services and strategies.to combat the spread of the two pandemics. India which has the highest TB global announced a Rapid Response Plan (RRP) to mitigate the impact of the COVID-19 Pandemic on the TB Epidemic includes bi-directional TB-Covid-19 screening and intensified case finding using geospatial trackers on TB, and COVID-19 to determine the geographic area needed to be targeted (39, 40). This is in concordance with our study and thus such tools can be applied to upgrade and boost the efforts to combat TB and other infectious diseases.

Geographic and spatiotemporal mapping for HIV/HCV coinfection has been found an important tool in highlighting the most prevalent geographic areas that need to be targeted (41). This has been applied in New Haven, Connecticut-USA to find that HIV/HCV coinfection status in an urban Northeast setting is distinct and has important implications for surveillance, healthcare delivery, disease prevention, and clinical care (42, 43). In our study, the HIV/HCV coinfection is predominantly evident in the Southern region area particularly Benghazi and Derna followed by Musrata in the Western region followed by Zawra and Jufra municipalities. Recent studies carried out on the impact of armed conflict of HIV/HCV in Ukraine and Libya have shown that viral dissemination changes were observed within the country (44, 45). A major virus flows from the Eastern region during the armed conflict associated with internally displaced people in Libya. Hence then syndemic geographic profiling becomes a necessity to trace the hot spots that need special attention in prevention and management.

Mortality and morbidity, among civilians and soldiers, are undoubtedly the primary indicators for assessing the severity and disruptiveness of warfare in human societies (46). In studying the systematic geographic profiling of morbidity and mortality, we efficiently visualized the patterns of mortality and morbidity during the Libyan conflict. The analysis showed that certain districts have been plagued by the conflict for a longer period. Spatial variation is noticeable within these regions, particularly Derna, Ajdabia, and Benghazi, followed by Sert, Musrata, Tripoli, and Jufra. The heaviest clusters of conflict-related mortality were in Benghazi and Derna in the east of the country and Sert in the central region. Hence then special intervention policy should be directed to such affected regions.

Our study indicates that there are important opportunities to closely examine districts and regions with notably high rates of integrated diseases such as TB/COVID-19, HIV/HCV, and mortality/morbidity. Such information is an important input for developing an effective public health response policy to combat the consequences of the conflict (47). Hence then, developing integrated pathways based on this approach becomes a priority in tackling multi-integrated diseases, particularly during conflict timing.

### 4.1 Limitations

Despite this study highlights a new and important finding, it is however, limited by number of factors. Including the number of the studied cases may not represent the accurate numbers of affected patients as it is difficult to control and get the accurate information during the armed conflicts. Geographic mapping particularly during COVID-19 and ongoing war periods show levels of uncertainty (standard errors), often based on very small numbers and, therefore, interpretation should be done cautiously. The study did not consider another integrated infectious and metabolic diseases and hence then further studies are needed. Notwithstanding such limitations, this showed the number of high risk individuals who are affected and thus allowed us to use a real-world patient population to assess screening and treatment priorities for communities with markedly increased health disparities, particularly HIV/HCV and TB/COVID-19 coinfection and other similar infections.

## 5 Conclusion

To the best of our knowledge, this study represents the first attempt to disaggregate the syndemic geographic patterns of certain integrated diseases during the armed conflict. This approach could offer valuable public health tools that can be applied to upgrade and boost the efforts to combat co-occurring diseases. Taken together, these findings confirm interactions among individuals and geographic areas with s syndemic geographic problems which needed to examined at a regional and national particularly during and after the war conflict ended (48, 49).

## Data Availability

The original contributions presented in the study are included in the article/Supplementary Material, further inquiries can be directed to the corresponding author/s.
